# EHD2 is a Predictive Biomarker of Chemotherapy Efficacy in Triple Negative Breast Carcinoma

**DOI:** 10.1038/s41598-020-65054-5

**Published:** 2020-05-14

**Authors:** Wei-Wei Shen, Ivan Bièche, Laetitia Fuhrmann, Sophie Vacher, Anne Vincent-Salomon, Stéphanie Torrino, Christophe Lamaze

**Affiliations:** 1 Institut Curie - Centre de Recherche, PSL Research University, Membrane Dynamics and Mechanics of Intracellular Signaling team, 75248 Paris, cedex 05 France; 2 Institut National de la Santé et de la Recherche Médicale (INSERM), U1143 Paris, France; 3 Centre National de la Recherche Scientifique (CNRS), UMR, 3666 Paris, France; 4 Pharmacogenomics Unit, Department of Genetics, Institut Curie, 26 rue d’Ulm, 75248 Paris, cedex 05 France; 5 Department of Pathology, Institut Curie, 26 rue d’Ulm, 75248 Paris, cedex 05 France; 6 Institut Curie, PSL Research University, INSERM U830, 26 rue d’Ulm, 75248 Paris, cedex 05 France; 7 CNRS UMR7275, Institut de Pharmacologie Cellulaire et Moléculaire, Université Côte d’Azur, 06560 Valbonne, France

**Keywords:** Cancer, Breast cancer

## Abstract

EHD2 is a mechanotransducing ATPase localized in caveolae invaginations at the plasma membrane. EHD2 has recently been associated with several human cancers, however the significance of EHD2 transcript levels in cancer prognosis remains debated. Breast cancer is the most commonly occurring cancer in women and prognosis is variable depending on the subtypes. Triple negative breast cancer (TNBC) often has a poor therapeutic response. The aim of this study was to assess the prognostic significance of EHD2 transcripts and protein expression levels in breast carcinomas. We found that low EHD2 levels were associated with enhanced proliferation, migration and invasion of TNBC cells. EHD2 expression was significantly reduced in TNBC tissues and the loss of EHD2 led to higher expression of the pro-tumoral cytokine IL-8. In apparent contradiction with *in vitro* data, multivariate analysis of two independent cohorts of breast cancer patients revealed that low EHD2 was in fact associated with good prognosis in the highly proliferative TNBC subtype. Accordingly, TNBC low EHD2 expressers were found to benefit the most from chemotherapy when compared to all subtypes of breast cancers. Our study validates EHD2 expression level as an independent prognostic factor of metastasis-free survival and as a new predictive marker of chemotherapy efficacy in TNBC patients.

## Introduction

Eps15 homology domain containing protein 2 (EHD2) is a cytosolic ATPase localized at the neck of small plasma membrane invaginations called caveolae, where it controls their stability and dynamics at the cell surface^1,2^. We recently established EHD2 as a key element in mechanotransduction by connecting caveolae mechanosensing with gene transcription under mechanical stress^3^. EHD2 has recently been associated with several types of cancer. It has been shown that EHD2 protein levels were reduced in esophageal squamous cell carcinoma compared to normal tissues^4^. Low expression of EHD2 increased the migration of human esophageal squamous cell carcinoma TE1 cells^4^ and similar results have also been reported in ovarian cancer, malignant melanoma and hepatocellular carcinoma^5–7^. In line with these findings, it was also shown that EHD2 can inhibit metastasis of breast cancer^8,9^.

Breast cancer is the most commonly occurring cancer and the number one cause of cancer mortality in women in the world^10^. Breast cancer prognosis is variable, depending mostly on the molecular features of the tumor and on tumor stage at diagnosis. Breast tumors can be separated into different molecular subtypes: (1) the luminal A and B subtypes, overexpressing progesterone (PR) and/or estrogen receptors (ER), (2) the HER2 + subtype, expressing high levels of the human epidermal growth factor receptor 2 (HER2) protein and (3) the triple negative breast cancers (TNBC) expressing no PR and/or ER and without HER2 overexpression and/or amplification^11–13^. Although TNBC account for only 15% of primary breast cancers^14^, they remain an important therapeutic challenge because of their poor prognosis, with variable responses to chemotherapy^15,16^. An important challenge is therefore to identify new targets and associated biomarkers for TNBC therapy. In this study, we found that the expression of EHD2 was significantly reduced in TNBC as compared to the other subtypes of breast cancers, and that the loss of EHD2 significantly enhanced the migration and the invasion of TNBC cells. Moreover IL-8, a cytokine endowed with a pro-tumorigenic function, was negatively regulated by EHD2. Low EHD2 expression was associated with good prognosis in TNBC in two independent cohorts. Finally, TNBC low EHD2 expressers showed a positive response to chemotherapy compared with all subtypes of breast cancers. Altogether, our data establish EHD2 as a tumor suppressor which is an independent prognostic factor of metastasis-free survival and a new predictive biomarker of chemotherapy efficacy in TNBC.

## Results

### EHD2 expression is significantly reduced in triple negative breast cancer tissues

We examined EHD2 mRNA expression in a retrospective cohort of 526 breast cancer patients that was collected over the last 30 years (Supplementary Table S1). This allowed us to determine if variations in the mRNA expression of EHD2, measured by quantitative RT–PCR, corresponded with patient prognosis. In this cohort, we observed an overall under-expression of EHD2 mRNA of 31.2%, defined as <0.33 relative to normal. We next analyzed EHD2 expression with the standard clinicopathological parameters (Table 1). We found significant associations between low EHD2 transcript expression levels and high histological grade (grade III), increased cell proliferation (as determined by the expression of proliferation marker Ki67), positive lymph nodes, high macroscopic tumor size, and ER-negative and PR-negative status, as well as with TNBC sub-type (Table 1). These results, obtained at the RNA level, establish that low RNA expression of EHD2 in breast cancer patient tissues is associated with high breast cancer aggression.

**Table 1:** Relationship between EHD2 transcript levels and standard clinicopathological parameters in a series of 526 breast cancers.

	Total population (%)	Number of patients (%)		*p*^a^
		EHD2 mRNA expression <0.33 relative to normals	EHD2 mRNA expression >=0.33 relative to normals	
Total	526 (100)	164 (31.2)	362 (68.8)	
***Age***				
≤50	124 (23.6)	43 (26.2)	81 (22.4)	0.34 (NS)
>50	402 (76.4)	121 (73.8)	281 (77.6)	
***SBR histological grade***^***b,c***^				
I	60 (11.7)	7 (4.4)	53 (15.1)	**0.0000021**
II	240 (47.0)	61 (38.4)	179 (50.9)	
III	211 (41.3)	91 (57.2)	120 (34.1)	
***Macroscopic tumor size***^***e***^				
≤25 mm	247 (47.9)	63 (39.1)	184 (51.8)	**0.0075**
>25 mm	269 (52.1)	98 (60.9)	171 (48.2)	
***Lymph node status***^***d***^				
0	159 (30.5)	66 (40.2)	93 (26.1)	**0.0041**
1–3	249 (47.8)	70 (42.7)	179 (50.1)	
>3	113 (21.7)	28 (17.1)	85 (23.8)	
***ERα status***				
Negative	181 (34.4)	79 (48.2)	102 (28.2)	**0.0000078**
Positive	345 (65.6)	85 (51.8)	260 (71.8)	
***PR status***				
Negative	255 (48.5)	107 (65.2)	148 (40.9)	**0.00000022**
Positive	271 (51.5)	57 (34.8)	214 (59.1)	
***HER2 status***				
Negative	396 (75.3)	124 (75.6)	272 (75.1)	0.91 (NS)
Positive	130 (24.7)	40 (24.4)	90 (24.9)	
***Molecular subtypes***				
HR− HER2−	102 (19.4)	52 (31.7)	50 (13.8)	**0.000012**
HR− HER2+	72 (13.7)	26 (15.9)	46 (12.7)	
HR+ HER2−	294 (55.9)	72 (43.9)	222 (61.3)	
HR+ HER2+	58 (11.0)	14 (8.5)	44 (12.2)	
***KI67 mRNA expression***^***f***^				
median	12.5 (0.80–313)	14.9 (2.73–313)	11.6 (0.80–94.5)	**0.00036**^**g**^

Expression of EHD2 was examined in a series of breast tumors from 526 patients and in 16 normal breast tissues. mRNA values were quantified using RT–qPCR. Values of breast cancer samples were normalized to the median of the 16 normal breast tissue values.

^a^χ^2^Test.

^b^Scarff Bloom Richardson classification.

^c^Information available for 511 patients.

^d^Information available for 521 patients.

^e^Information available for 516 patients.

^f^Information available for 447 patients.

^g^Kruskal Wallis’s H Test.

**Figure 1:**
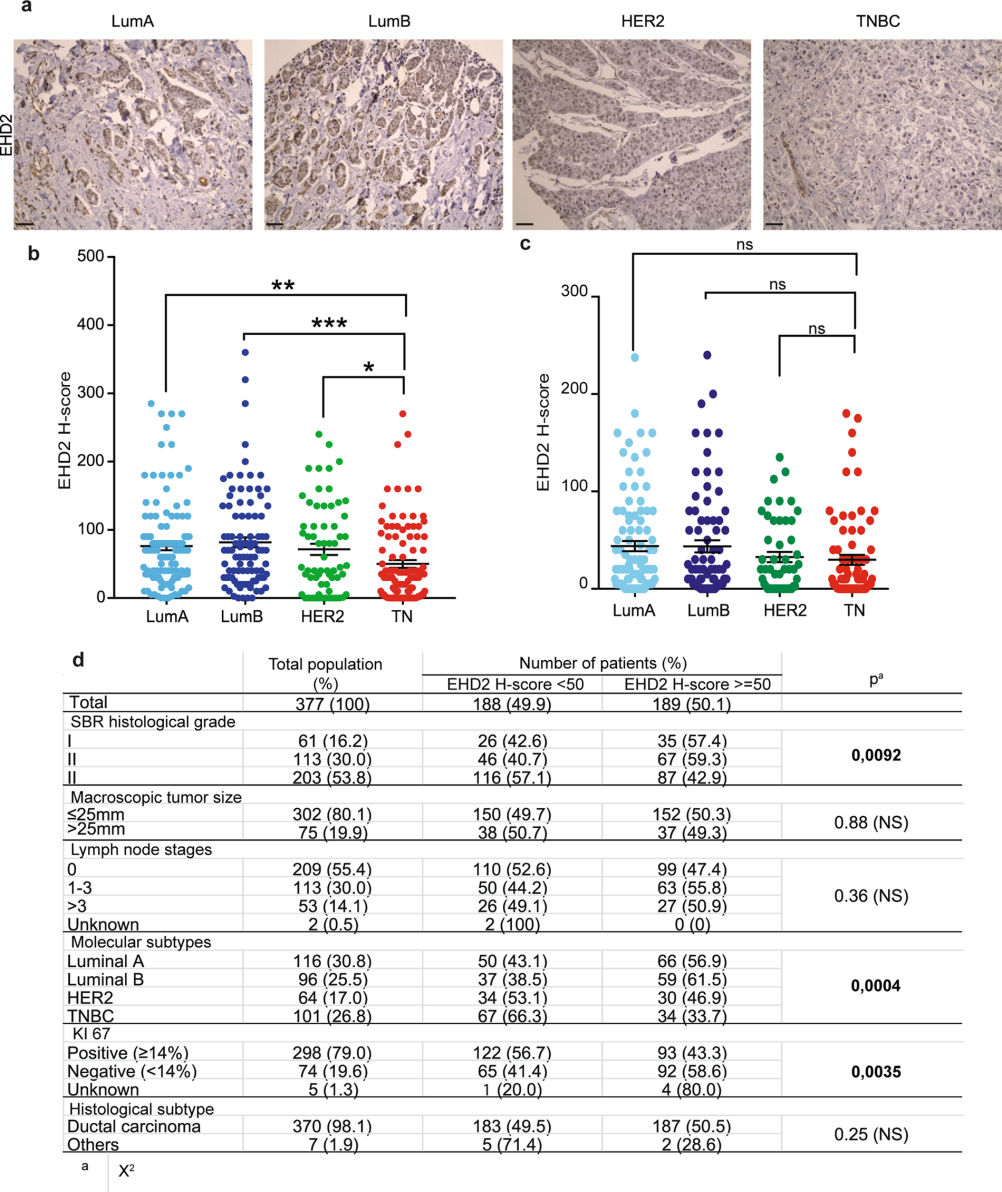
Low EHD2 expression is associated with TNBC patients. (**a**) Representative EHD2 immunohistochemistry staining on sections of human breast tumor (TMA). Scale bar = 100 μm. (**b**) Quantification of EHD2 H-score (immunohistochemistry intensity multiplied by percentage of positively stained cells) in luminal, HER2+ and TNBC tissues. (**c**) Quantification of EHD2 H-score (immunohistochemistry intensity multiplied by percentage of positively stained cells) in stroma cells of luminal, HER2+ and TNBC tissues. (**d**) EHD2 protein levels and standard clinicopathological parameters in a series of 377 breast cancers (TMA). Underexpressed EHD2 corresponds to EHD2 H-score <50 whereas overexpressed EHD2 correspond to EHD2 H-score ≥50. ns = non-significant; *P < 0.05; **P < 0.01; ***P < 0.001; (**b**) Bonferroni’s multiple comparison test; mean ± s.e.m.

We confirmed these characteristics at the protein level in breast carcinoma biopsies from an independent cohort of patients. Immunohistochemical analysis of a tissue microarray (TMA) of 423 invasive breast tumors (Supplementary Table S2) revealed that EHD2 was localized in the nucleus of epithelial tumor cells and stromal cells i.e. fibroblasts, inflammatory, and endothelial cells (Fig. 1a). Scoring of staining intensity revealed that a low H-score of EHD2 expression was significantly associated with triple negative basal-like cancers, when compared to HER2 and luminal breast cancers (Fig. 1a,b). Importantly, EHD2 expression was not changed in the stromal cells surrounding the tumor (Fig. 1c). Significant associations were found between EHD2 and tumor grade, ER status, PR status, HER2 status and proliferation using Ki67 in this cohort of 421 patients (Fig. 1d). However, no significant associations were found between EHD2 and tumor size, lymph node status, and cancer histological subtype.

### The majority of triple negative breast cancer cell lines have low EHD2 expression

**Figure 2:**
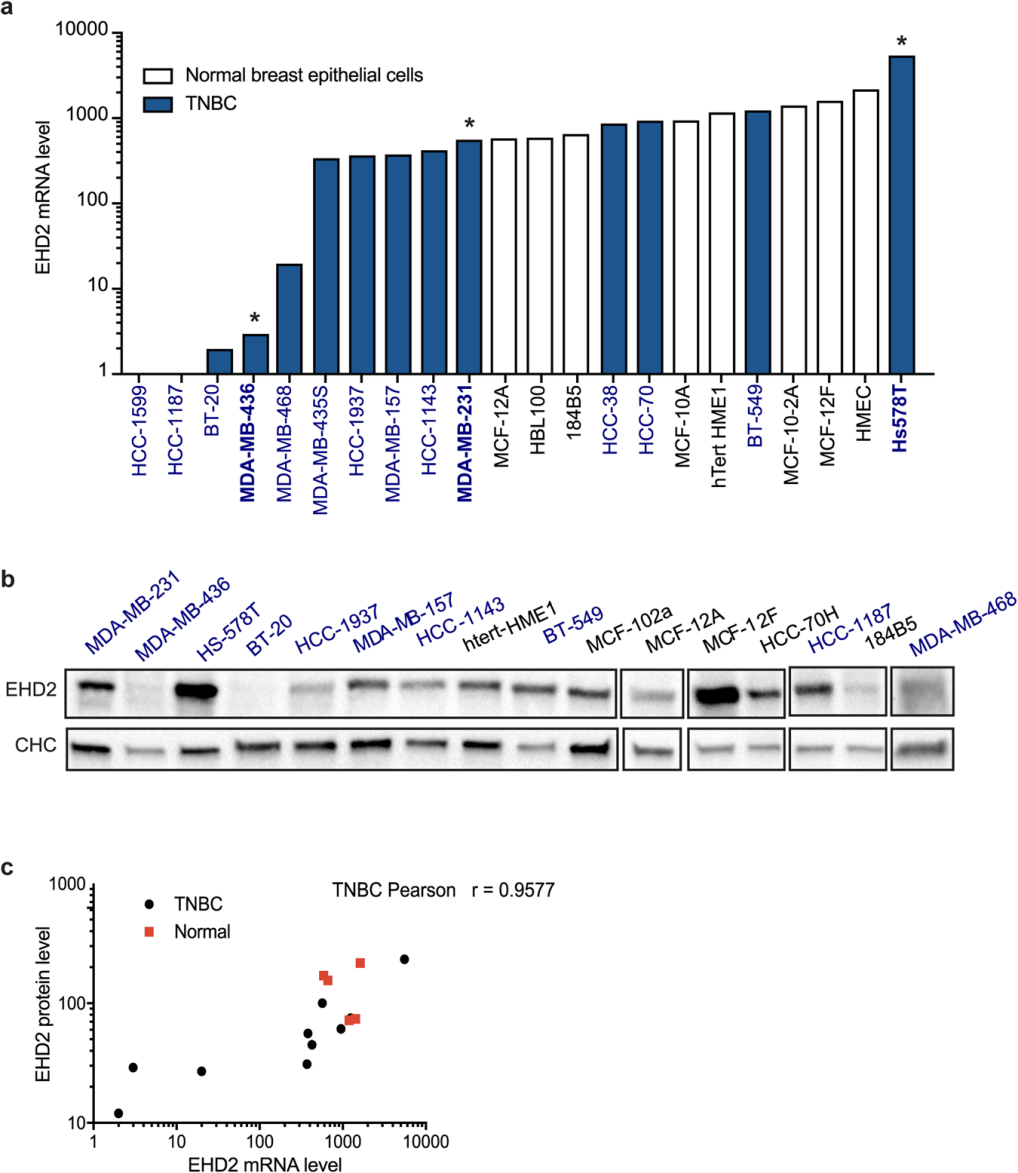
EHD2 expression in breast epithelial and cancer cell lines. (**a**) EHD2 mRNA levels in 22 breast cancer cell lines representing various human TNBC (blue) and normal breast tissue cell lines (white). *Indicates cell lines selected for experiments. (**b**) Immunoblot analysis for EHD2 expression in 16 breast cancer cell lines representing human TNBC and normal breast tissue cell lines. (**c**) The correlation between EHD2 mRNA and EHD2 protein in various TNBC and normal breast tissue cell lines. Pearson’s correlation coefficients for TNBC and normal breast cell lines were scored; *P < 0.05; (**c**) two tailed t-test; mean ± s.e.m.

Next, we analyzed EHD2 transcript expression in 22 breast-tissue derived cell lines representing various TNBC and normal breast epithelial cells. In alignment with our analysis on patient biopsies, measurement of EHD2 mRNA transcripts revealed that the majority (72%) of cell lines derived from triple negative basal-like breast cancers had low EHD2 mrna expression compared to non-cancer epithelial cell lines (Fig. 2a). We next sought to verify that EHD2 downregulation at the mRNA level also corresponded  to downregulation at the protein level (Fig. 2b). A high correlation was observed between EHD2 mRNA and protein levels in triple negative basal-like breast cancer cell lines as reflected by the Pearson’s correlation index (r = 0.95) (Fig. 2c).

### EHD2 downregulation promotes aggressiveness in breast cancer cells

To better understand the significance of variations of EHD2 expression in breast cancers, we selected the Hs578T, MDA-MB-231 and MDA-MB-436 TNBC cell lines that respectively express three representative levels of EHD2 transcripts i.e. high, medium, and low (Fig. 2a).

**Figure 3:**
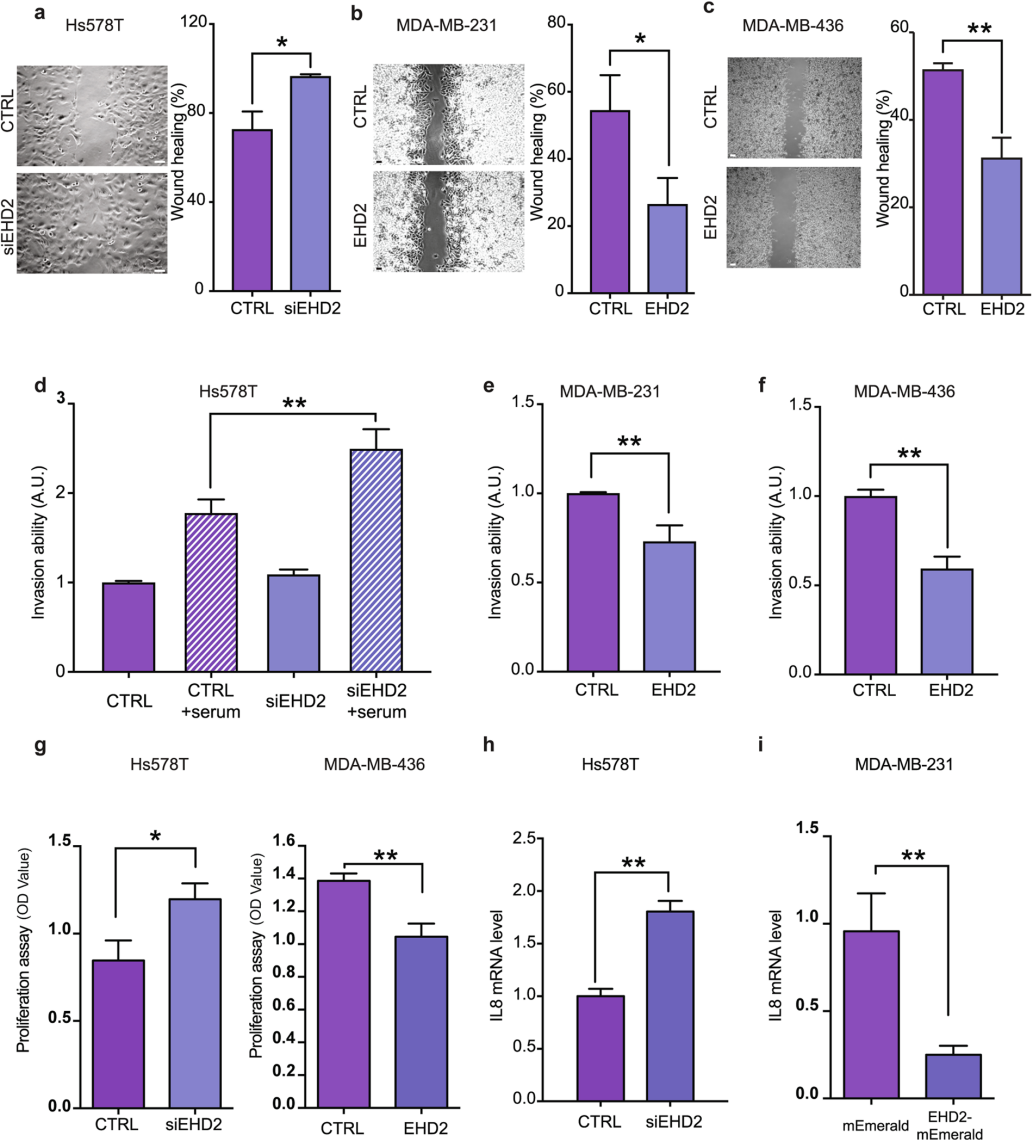
EHD2 downregulation is associated with breast cancer cell aggressiveness. (**a**–**c**) Representative transmitted light images (left) and quantification (right) of cell migration using a wound healing assay in Hs578T cells EHD2 depleted (siEHD2) or not (CTRL) (**a**), in MDA-MB-231 cells overexpressing EHD2 or not (CTRL) (**b**), and in MDA-MB-436 cells overexpressing EHD2 or not (CTRL) (**c**). Scale bar =10 μm. Cell migration into the wound site was assessed after 16 h. (**d–f**) Quantification of invasion using Transwell chamber inserts in Hs578T cells EHD2 depleted (siEHD2) or not (CTRL) in the absence (negative control) or presence of serum (**d**), in MDA-MB-231 cells overexpressing EHD2 or not (CTRL) (**e**), and in MDA-MB-436 cells overexpressing EHD2 or not (CTRL) (**f**). (**g**) Measurement of cell proliferation of the Hs578T cells EHD2 depleted (siEHD2) or not (CTRL), and in MDA-MB-436 cells overexpressing EHD2 or not (CTRL). (**h,i**) Quantification of IL-8 mRNA levels in Hs578T cells EHD2 depleted (siEHD2) or not (CTRL) (**h**), and in MDA-MB-231 cells overexpressing EHD2 (**i**); n ≥ 3 independent experiments; ns = non-significant; *P < 0.05; **P < 0.01; (**a–c,e–i**) two tailed t-test; (**d**) Bonferroni’s multiple comparison test; mean ± s.e.m.

We used an established *in vitro* cell migration/wounding assay to investigate the role of EHD2 in the aggressiveness of Hs578T, MDA-MB-231 and MDA-MB-436 TNBC cells that are respectively defined as moderately (Hs578T) and highly invasive (MDA-MB-231 and MDA-MB-436). As expected from the transcript levels, immunoblot analyses confirmed that Hs578T, MDA-MB-231 and MDA-MB-436 TNBC cell lines express high, medium and low levels of EHD2 protein, respectively (Fig. 2a,b). EHD2 depletion enhanced Hs578T cell migration (Fig. 3a), whereas overexpression of EHD2 reduced the migratory activity of MDA-MB-231 and MDA-MB-436 cells (Fig. 3b,c). We next analyzed the invasive capacity of these cells using a Transwell assay, and again found that invasion was dependent on EHD2 expression, as it was increased by EHD2 depletion in Hs578T cells and reduced by overexpression of EHD2 in MDA-MB-231 and MDA-MB-436 cells (Fig. 3d–f). Moreover, proliferation was increased by EHD2 depletion in Hs578T cells and reduced by overexpression in MDA-MB-436 cells (Fig. 3g). Interestingly, DNA microarrays revealed that the mRNA transcript levels of the inflammatory cytokine interleukin-8 (IL-8) was inversely correlated with the level of EHD2 transcripts in Hs758T cells (data not shown). IL-8 is known to be upregulated in several cancers, including breast cancer, where it controls several parameters involved in cancer progression including cell migration and invasion. More recent evidence indicates that this cytokine is also a key regulator of cancer stem cell activity^17,18^. We confirmed *IL-8* up-regulation in Hs578T cells upon EHD2 depletion, whereas *IL-8* was downregulated in MDA-MB-231 upon EHD2 overexpression (Fig. 3h,i). These results suggest that the loss of EHD2 promotes IL-8 levels in breast cancer cell lines. Altogether, these findings establish EHD2 mRNA and protein expression levels as key parameters in the control of breast cancer cell migration and invasion.

### Low EHD2 expression is associated with good prognosis in triple negative breast cancer

**Figure 4:**
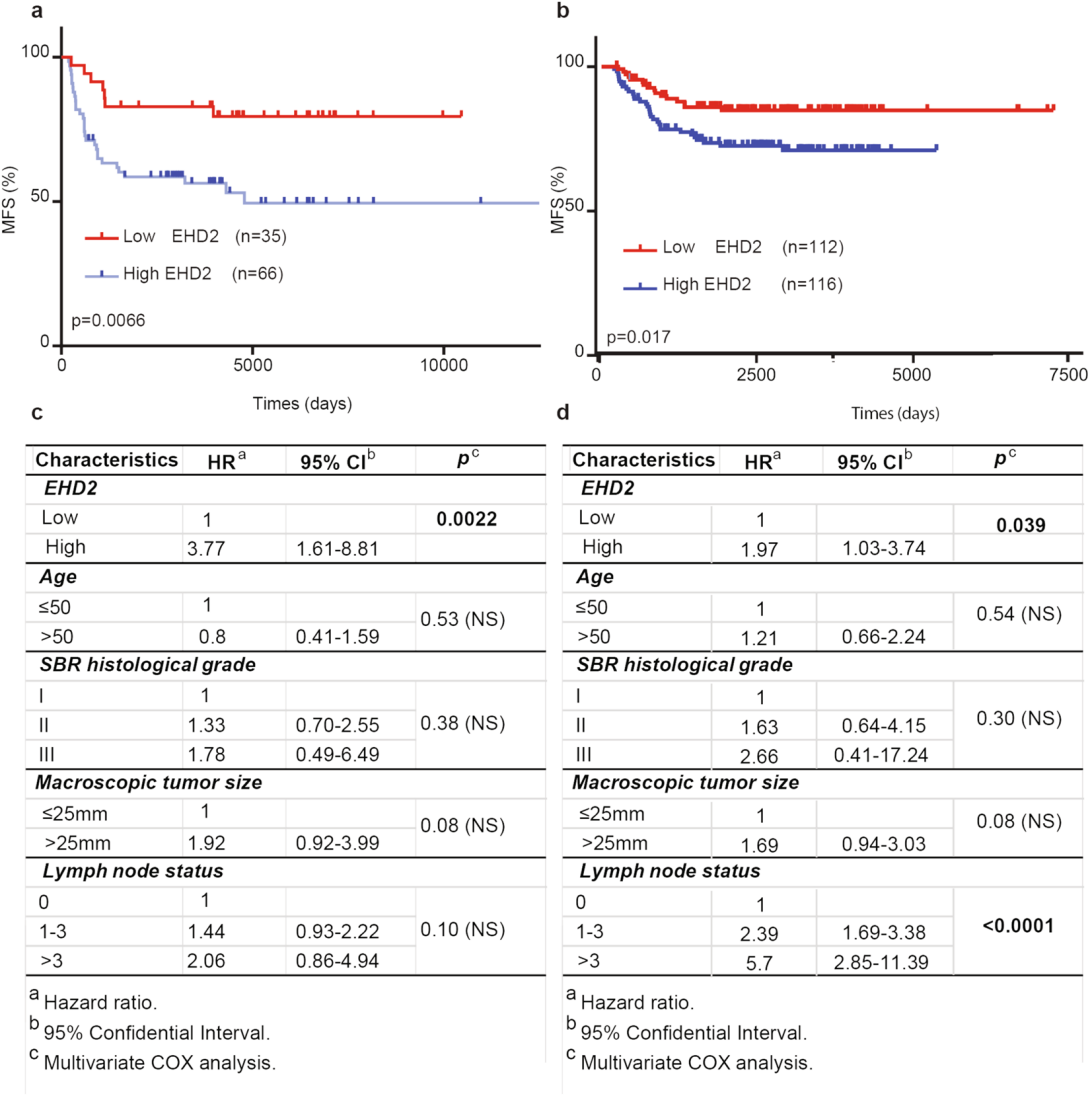
Low EHD2 mRNA expression is associated with good prognosis in TNBC and is a new independent prognostic factor of MFS. Analysis of MFS in (**a**) 101 or (**b**) 228 patients with TNBC was determined as the interval between initial diagnosis and detection of the first metastasis. Survival distributions were plotted using the Kaplan–Meier method, and the significance of the difference was ascertained with the log-rank test using optimal cutoffs (**a**: 0.18, **b**: 0.15). Patients with high EHD2 mRNA expression have a significantly poorer prognosis compared with patients with reduced EHD2 mRNA expression (**a**: *P* = 0.0066, **b**: *P* = 0.017). (**c**) Multivariate COX analysis of MFS for EHD2 mRNA expression in the series of (**c**) 101 or (**d**) 228 patients with TNBC.

We next analyzed a cohort of 101 TNBC patients, from the cohort of the 526 patients for metastasis-free survival (MFS) (Supplementary Table S3). The median follow-up time of these patients was 10 000 days (around 30 years). In apparent contradiction with the data obtained in cell lines, we found that low levels of *EHD2* transcripts were significantly associated with increased MFS (Fig. 4a; p = 0.0066). Indeed, in patients with reduced *EHD2* expression, there was 80% MFS *vs*. 50% for patients with high *EHD2* expression. To confirm our results, we also analyzed MFS for a second independent cohort of 228 patients with TNBC (Supplementary Table S4). *EHD2* RNA downregulation was also associated with increased MFS in a statistically significant manner (Fig. 4b; p = 0.017). Multivariate analysis using a Cox proportional hazard model assessed the predictive value for MFS of the significant parameters on univariate analysis, that is, tumor size, SBR histological grade, lymph node status, age and EHD2 low or high expression. The prognostic significance of EHD2 compared to the other parameters indicate that EHD2 status represents an independent prognostic factor of MFS (Fig. 4c,d).

### EHD2 expression levels predict chemotherapy efficacy in TNBC

**Figure 5:**
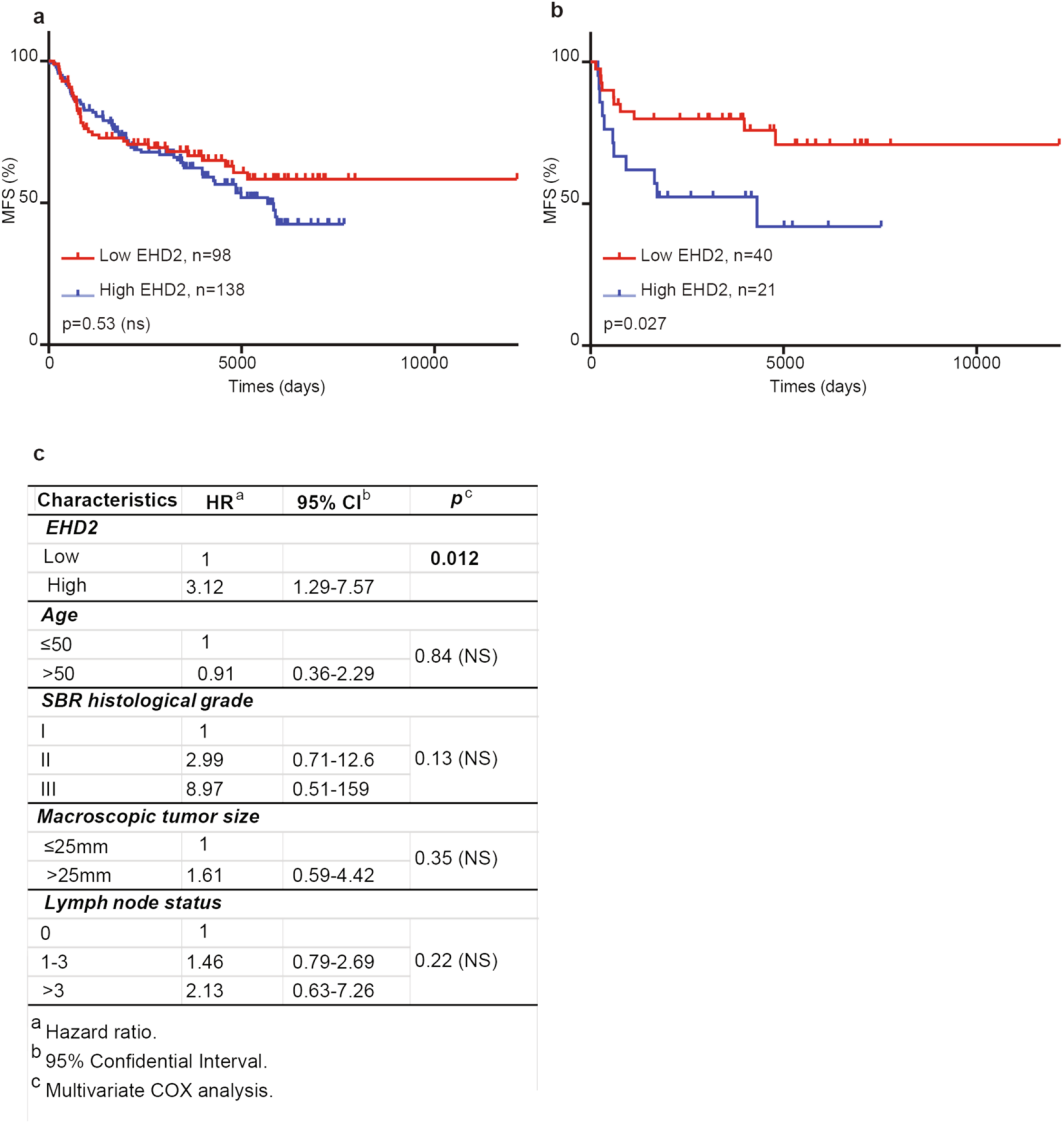
EHD2 expression levels predict chemotherapy efficacy in TNBC. (**a**) Analysis of MFS in 236 patients treated with chemotherapy was determined by the interval between initial diagnosis and detection of the first metastasis. Survival distributions were plotted using the Kaplan–Meier method, and the significance of the difference was ascertained with the log-rank test using optimal cutoffs (0.385). (**b**) MFS of 61 patients with TNBC treated with chemotherapy was determined by the interval between initial diagnosis and detection of the first metastasis. Survival distributions were plotted using the Kaplan–Meier method, and the significance of the difference was ascertained with the log-rank test using optimal cutoffs (0.3955). Patients with high EHD2 mRNA expression had a significantly poorer prognosis compared to patients with reduced EHD2 mRNA expression (*P* = 0.027). (**c**) Multivariate COX analysis of MFS for EHD2 mRNA expression in the series of 61 patients with TNBC treated with chemotherapy.

Next, from the cohort of the 526 patients for MFS, we specifically analyzed a cohort of 236 patients treated with chemotherapy (Supplementary Table S1). The median follow-up time of these patients was 10 000 days (around 30 years). We found that levels of *EHD2* transcripts were not associated with increased MFS (Fig. 5a). We also analyzed a cohort of 61 TNBC patients treated with chemotherapy taken from the cohort of 101 TNBC patients for MFS (Supplementary Table S3). Interestingly, we found that low levels of *EHD2* transcripts were significantly associated with increased MFS (Fig. 5b; p = 0.027) Multivariate analysis using a Cox proportional hazard model assessed the predictive value for MFS of the significant parameters on univariate analysis, that is, tumor size, SBR histological grade, lymph node status, age and EHD2 low or high expression. The prognostic significance of EHD2 compared to the other parameters indicate that EHD2 status is also an independent prognostic factor of MFS in TNBC treated with chemotherapy (Fig. 5c). Altogether, these findings established EHD2 expression level as predictive marker for MFS and chemotherapy efficacy in TNBC.

## Discussion

In this study, we analyzed the significance of mRNA and protein levels of EHD2 in breast cancer prognosis. In two distinct cohorts of TNBC patients, we found that EHD2 downregulation was associated with triple negative breast cancer and was a new independent prognostic factor of MFS. Downregulation of EHD2 was also observed in the majority of TNBC derived cell lines both at mRNA and protein levels. The EHD protein family is involved in intracellular trafficking, including endocytosis and cargo recycling at the plasma membrane^19^. EHD2 is a cytosolic ATPase that is only found in caveolae, specialized plasma membrane invaginations classically involved in membrane trafficking and signaling^20^. EHD2 localization is restricted to the neck of caveolae where it controls their stability at the plasma membrane^1,2,21^. We recently discovered a new role for EHD2 as a central player in mechanotransduction by connecting the caveolae response to mechanical stress with the regulation of gene transcription^3^. EHD2 has recently drawn attention to its potential role in cancer development. The first genomic study, performed by Santin and colleagues in 2006, identified EHD2 as being downregulated 15-fold in ovarian serous papillary carcinoma in comparison with control human ovarian surface epithelium^5^. Decreased EHD2 expression was also reported to enhance migration of human breast cancer cells^8^, a function that has been related to Rac1 and E-cadherin expression^9^.

Here, we examined EHD2 expression in breast cancer cell lines and breast cancer patients. We observed a negative correlation between EHD2 expression and tumor aggressiveness, especially in TNBC patients. We found that the decrease of EHD2 expression was correlated with enhanced proliferation, migration and invasion of triple negative breast cancer cells. These data obtained in two distinct cohorts of TNBC patients establish EHD2 as a new independent prognostic factor of MFS.

Changes in EHD2 expression levels revealed a correlation with IL-8 expression in breast cancer cell lines. This new correlation between EHD2 and IL-8 levels may explain the anti-metastatic role of EHD2 in cancer. Indeed, IL-8 is known to be upregulated in several types of solid cancers, such as prostate, gastric, bladder, ovarian and lung cancers, and has been associated with different hallmarks of cancer progression, such as increased proliferation, angiogenesis, invasion and metastasis formation^22–24^. While there is substantial evidence that IL-8 may induce breast cancer initiation and progression via the above mechanisms, more recent evidence indicates that this cytokine is also an important regulator of cancer stem-like cell activity^25^. Our study is therefore the first one to report that the regulation of IL-8 may be regulated by EHD2, suggesting that EHD2 could be a potential target to inhibit the IL-8 pathway and cancer stem cell activity. Follow up experiments will be needed to further establish this correlation.

Downregulation of EHD2 has been associated with poor prognosis in esophageal squamous cell carcinoma, hepatocellular carcinoma and breast cancer^4,7,9^. In contrast, EHD2 overexpression was associated with poor prognosis in thyroid carcinoma^26^. We found in our study that low EHD2 expression is associated with good prognosis of TNBC. This unexpected conclusion may come from differences in the cohorts studied since Shi and colleagues analyzed one cohort of 96 patients with different subtypes of breast cancers, whereas we analyzed two independent cohorts of TNBC. Further studies will be required to better understand the role that EHD2 plays in various cancer types. While caveolae have long been involved in tumorigenesis, albeit with some controversy, our data confirm that the cell machinery associated with the response to mechanical forces can be deregulated in cancers^27^. It will be important to integrate this new mechanical aspect of EHD2 function in the future studies on its dysregulation in solid cancers.

TNBC can be divided into different subtypes: Basal-like 1 and 2 (BL1-BL2); Mesenchymal (M); Mesenchymal stem–like (MSL); Immunomodulatory (IM), and Luminal androgen receptor (LAR). It has been shown that TNBC subtypes can respond differentially to treatments^28^. Lehmann and colleagues identified and selected some preclinical models representing the different TNBC subtypes^28^. Indeed, our data indicate that low EHD2 expression correlated with the BL subtype whereas high EHD2 expression correlated with the MSL subtype. However, we found that MDA-MB-436, that are classified as MSL, also expressed low EHD2. It will be therefore important in future experiments to determine if EHD2 expression is associated to a specific subtype of TNBC.

The fact that low EHD2 expression in the TNBC subtype is associated with good prognosis, but also with an increase in cell proliferation, migration and invasion, may be seen as an apparent contradiction. It is nevertheless known that highly proliferative breast cancer cells respond better to chemotherapy^29^. Accordingly, we found that low EHD2 was a new independent prognostic factor in the subgroup of TNBC patients treated with chemotherapy compared to all other breast cancer subgroups. These results not only establish EHD2 as a metastatic suppressor, they further define EHD2 as a new independent prognostic factor of metastasis-free survival that can be used as a biomarker to predict chemotherapy efficacy in TNBC.

## Conclusions

In this study, we have found that low EHD2 expression in TNBC cells correlated with enhanced proliferation, migration and invasion *in vitro*. However, analysis of TNBC patient samples revealed that low EHD2 was predictive of chemotherapy efficacy. We propose that the increased proliferation observed in TNBC cells with low levels of EHD2 allows a better therapeutic response since chemotherapy is more efficient on highly proliferative cells. Altogether, our study not only reveals that EHD2 acts as a tumor suppressor, it is also a new independent prognostic factor of metastasis-free survival and a new biomarker of chemotherapy response in TNBC patients.

## Methods

### Human sample analysis

Analyses of human samples were performed in accordance with the French Bioethics Law 2004–800, the French National Institute of Cancer (INCa) Ethics Charter and after approval by the Institut Curie review board and ethics committee (Comité de Pilotage du Groupe Sein). Women were informed of the research use of their tissues. Informed written consent was obtained from all participants after detailed explanation of the study. Data were analyzed anonymously.

### TMA

423 samples of primary breast tumors surgically removed before any radiation, hormonal or chemotherapy treatment at Institut Curie from 2005 to 2006 have been analyzed. 235 samples of luminal A and B, 83 samples of HER2 + and 105 samples of TNBC were analyzed in this study (Supplementary Table S2). all TNBC and HER2 tumors available and an equal number of consecutively treated luminal tumors were alcohol formalin acetic acid-fixed and paraffin-embedded. Breast molecular subtypes were defined as follows: luminal [luminal A, estrogen receptor (ER) ≥ 10%, progesterone receptor (PR) ≥ 20%, Ki67 < 14%; luminal B, ER ≥ 10%, PR < 20%, Ki67 ≥ 14%]^30^; ER − PR − HER2 + (HER2), ER < 10%, PR < 10%, HER2 2+ amplified or 3+; ER − PR − HER2 − (TNBC), ER < 10%, PR < 10%, HER2 0/1+ or 2+ nonamplified according to the American Society of Clinical Oncology (ASCO) guidelines^31^. TMA consisted of 1 mm diameter tumor core replicates selected from whole-tumor tissue sections in the most representative tumor areas (high tumor cell density) of each tumor sample and a matched tissue core from adjacent non-tumoral breast epithelium. For EHD2 immunohistochemistry, EnVision FLEX, High pH kit (Dako, Les Ulis, France) was used according to the manufacturer’s instructions^32^. Analyses were performed in a blinded manner with 3 independent observers. H-score (IHC intensity multiplied by the percentage of positively stained cells) in the different molecular subtypes of breast cancers were quantified.

### RT-qPCR in patients, samples and cell lines for EHD2 expression

We analyzed 526 samples of primary unilateral invasive breast tumors excised from women at the Institut Curie/René Huguenin Hospital (SaintCloud, France) from 1978 to 2008 (Supplementary Table S1). Samples were examined histologically for the presence of tumor cells. Immediately after biopsy or surgery, the tumor samples were collected and stored in liquid nitrogen until mRNA extraction. A tumor sample was considered suitable for this study if the proportion of tumor cells was more than 70%. Estrogen receptor (ER), progesterone receptor (PR), and human epidermal growth factor receptor 2 (HER2) status was determined at the protein level by using biochemical methods (dextran-coated charcoal method used until 1988 (ref34)), enzyme immunoassay (used from 1988 onwards [Abbott ER-EIA-monoclonal kit and Abbott PgR-EIA-monoclonal kit; Abbott Laboratories, North Chicago, IL]), or immunohistochemistry) and confirmed by real-time quantitative RT-PCR assays^33,34^, The detection cutoff was set at 10 fmol/mg protein until 1988 and then at 15 fmol/mg protein. The Triton c-erbB-2 Tissue Extract EIA kit (Ciba Corning Diagnostics, Alameda, CA) is a double monoclonal antibody-based assay. The two monoclonal antibodies, designated TAB 257 and TAB 259, are specific for different epitopes of the external domain of the c-erbB-2 molecule^35^.

The population was divided into four groups according to HR (ER and PR) and HER2 status (Supplementary Table S1). 101 patients of the population were TNBC and the standard prognostic factors of this series is reported in Supplementary Table S3.

We also analyzed by RT-qPCR an independent cohort of 228 TNBCs. The standard prognostic factors of this second tumor series are reported in Supplementary Table S4.

Specimens of adjacent control breast tissue from eight breast cancer patients and normal breast tissue from eight women undergoing cosmetic breast surgery were used as sources of normal RNA.

Twenty-two samples of breast tissue-derived cell lines were analyzed. These cell lines were obtained from the American Type Culture Collection (ATCC, Manassas, VA, USA) or from the German Resource Centre for Biological Material (DSMZ, Braunschweig, Germany). They were cultured as per conditions recommended by the suppliers. mRNAs were provided by the Transfer Department of the Curie Institute (Paris).

For qPCR, quantitative values were obtained from the cycle number (Ct value) at which the increase in the fluorescence signal associated with exponential growth of PCR products began to be detected by the laser detector of the ABI Prism 7900 sequence detection system (Perkin–Elmer Applied Biosystems, Foster City, CA), using PE biosystems analysis software according to the manufacturer’s instructions.

The precise amount of total mRNA added to each reaction mix (based on optical density) and its quality (i.e. lack of extensive degradation) are both difficult to assess. Therefore, we also quantified transcripts of the TBP gene (Genbank accession NM_003194) encoding the TATA box-binding protein (a component of the DNA-binding protein complex TFIID) as an endogenous RNA control and normalized each sample on the basis of its TBP content. We selected TBP as an endogenous control because the prevalence of its transcripts is moderate, and because there are no known TBP retropseudogenes (retropseudogenes lead to coamplification of contaminating genomic DNA and thus interfere with RT-qPCR, despite the use of primers in separate exons)^36^.

Results, expressed as N-fold differences in target gene expressions relative to the TBP gene (and termed “NTARGET”) were determined as NTARGET = 2^ΔCtsample^, where the ΔCt value of the sample was determined by subtracting the Ct value of the specific target gene from the Ct value of the TBP gene. The NTARGET values of the samples were subsequently normalized such that the median of the NTARGET values for the 16 normal breast tissues was 1 in breast tumors, and such that the value for the “basal mRNA level” (smallest amount of quantifiable target gene mRNA, Ct = 35) was 1 in cell lines. Values of 0.33 or less was considered to represent underexpression, of the EHD2 gene in our tumor sample series. We have previously used the same cutoff point for tumor gene underexpression^37^.

The primers for TBP and EHD2 were chosen with the assistance of the Oligo 6.0 program (National Biosciences, Plymouth, MN). We scanned the dbEST database to confirm the total gene specificity of the nucleotide sequences chosen for the primers and the absence of single nucleotide polymorphisms. The nucleotide sequences of the primers used were as follows: EHD2-U (5′-TTT GCG AAG ATT CAG CTG GAA CAT-3′) and EHD2-L (5′-GGC TTC AGC GAG TGA AAC TTG GT-3′) PCR product of 113 bp and TBP-U (5′-TGC ACA GGA GCC AAG AGT GAA-3′) and TBP-L (5′-CAC ATC ACA GCT CCC CAC CA-3′) PCR product of 132 bp. To avoid amplification of contaminating genomic DNA, one of the two primers was placed at the junction between two exons. Agarose gel electrophoresis was used to verify the specificity of PCR amplicons. The conditions of total RNA extraction, complementary DNA synthesis and PCR were as previously described^33^.

### Cell culture

All cell lines were obtained from the American Type Culture Collection (ATCC; www.atcc.org) and cultured according to recommended conditions. All cells used in this study were within 10 passages after thawing. All cell lines were *Mycoplasma* free.

### RNA interference

Cells were transfected with siRNAs using HiPerFect (Qiagen) according to manufacturer’s instructions and cultured for the next 48 hours. Experiments were performed upon validation of depletion efficiency by immunoblot analysis using specific antibodies and normalizing to the total level of clathrin heavy chain (CHC) used as control. Control siRNA (SI03650325 5′-AATTCTCCGAACGTGTCACGT) was purchased from Qiagen and served as reference point. The EHD2 siRNA sequences were used at the final concentration of 20 nM (Qiagen, SI04205271 and SI04315108).

### Antibodies and plasmids

The following antibodies were purchased from the indicated suppliers: mouse monoclonal anti-EHD2 (Santa Cruz Biotechnology, sc-100724, 1:300 for western blotting and 1/50 for immunostaining); mouse monoclonal anti-clathrin heavy chain (BD Biosciences, 610500, 1:5,000 for western blotting); mouse monoclonal anti-GFP (Roche, 1:5,000 for western blotting); secondary antibody conjugated to horseradish peroxidase (HRP) (Beckman Coulter and Invitrogen).

Cells were transfected with EGFP or EHD2-EGFP using Lipofectamine 2000 (Invitrogen, Life Technologies) according to manufacturer’s instructions. Experiments were performed 6–24 hours after transfection. pEGFP was purchased from Clontech. EHD2-EGFP was generously provided by Dr. A. Helenius (ETH Zurich, Switzerland). pmEmerald was purchased from Addgene. EHD2-mEmerald was prepared by amplifying EHD2 from EHD2-EGFP using primers: F:AAAAAAAAGCTTCGATGTTCAGCTGGCTGAAGCGG; R: AAAAAAGGATCCCGCTCGGCGGAGCCCTTGT.

The product was inserted into pmEmerald using HindIII and BamHI enzymes. pmEmerald and EHD2-mEmerald plasmids were electroporated using Ingenio electroporation kit (Mirus) following manufacturer’s instructions. Electroporation of cells was performed with a pulse at 220 V and 975 μF with a Gene Pulser® II module (Bio-Rad). Experiments were performed 24-48 hours after transfection.

### Wound healing assay

Wound healing assays were performed using specific wound assay chambers (Ibidi). Cells were trypsinised and resuspended, 70 μl of cell suspension (5 × 10^5^ cells/ml) was seeded into each well of the insert. After allowing cell attachment for 24 hours, the culture inserts were removed, and the cells were incubated with fresh culture medium. Images of the cell migration into the defined cell free gap (500 μm width) were captured after 16 hours using Nikon phase contrast 2. Cells tracks were analyzed using the manual tracking software component of the Icy program.

### Migration and invasion assays

Transwell chambers were assembled using a 8 µm pore Thincert insert (Greiner Bio-One) as upper chambers and 24-well plates as lower chambers. For the invasion assay, cell culture inserts were coated with 100 µl Matrigel (1:1 diluted with PBS). Experiments were performed according to the manufacturer’s protocol. Briefly, cells were harvested, washed in PBS, and resuspended in serum free DMEM medium with 0.2% BSA. 600 µl of serum-free or complete DMEM medium with 0.2% BSA was added to the lower chamber. 200 µl of the cell suspension was added into the insert and incubated for 20 hours at 37 °C and 5% CO2. Subsequently, the medium was removed and replaced with 450 µl DMEM containing 0.2% BSA and 8 µM Calcein-AM. The plate with inserts was incubated for 45 min at 37 °C and 5% CO2. The cells were then detached using 600 µl of Trypsine-EDTA. Finally, migratory cells were quantified with a fluorescence plate reader (FLUOstar Optima, BMG) at an excitation wavelength of 485 nm and an emission wavelength of 520 nm.

### Cell proliferation assay

24 hours after electroporation or 48 hours after siRNA transfection, cells were seeded at 5 × 10^4^ cells/well in a 96-well plate and cultured for 24 hours prior to the experiment. All conditions were performed in triplicate. Cells transfected with pmEmerald or control siRNA served as control. Proliferation was measured using the Cell Proliferation ELISA BrdU kit (Roche Diagnostics GmbH), according to the manufacturer’s instructions. Briefly, cells were labeled with BrdU at a final concentration of 10 *μ*M/well, for 12 h at 37 °C. The cells were then denatured with FixDenat solution and incubated for 120 min with 1:100 diluted mouse anti-BrdU conjugated to peroxidase. After two washes (PBS 1×), the substrate solution was added for 25 min and, after this period, the reaction was stopped with 1 M H_2_SO_4_ solution. Absorbance was measured within 5 min at 450 nm with a reference wavelength at 690 nm using an ELISA plate reader.

### Statistical analysis

All analyses were performed using Prism 6.0 software (GraphPad Inc.). A two-tailed t-test was used when two conditions were compared. For more than two conditions, one-way ANOVA was used with Bonferroni’s multiple comparison test or Dunnett’s multiple comparison test (if comparing all conditions to the control condition). Significance of mean comparison is marked on the graphs by asterisks. Error bars denote SEM.

The correlation between mRNA expression of *EHD2* and the clinical parameters was assessed by nonparametric tests, chi-squared test (correlation between two qualitative parameters) and the Kruskal-Wallis H test (correlation between one quantitative parameter and one or more qualitative parameters). Differences were considered significant at confidence levels greater than 95% (p < 0.05). To visualize the efficacy of a molecular marker to discriminate between two populations (patients that developed/did not develop metastases) in the absence of an arbitrary cut-off value, data were summarized in a ROC (receiver operating characteristic) curve^38^. The AUC (area under curve) was calculated as a single measure to discriminate efficacy. Survival distributions were estimated by the Kaplan-Meier method, and the significance of differences between survival rates were ascertained with the log-rank test: Cox’s proportional hazard regression model was used to assess prognostic significance and the results are presented as hazard ratio and 95% confidence intervals. Metastasis-free survival (MFS) was determined as the interval between initial diagnosis and detection of the first metastasis.

## Supplementary information

Supplementary Information.

## Data Availability

Data is held within Curie Institute and is available on application.

## References

[1] Stoeber M (2012). Oligomers of the ATPase EHD2 confine caveolae to the plasma membrane through association with actin. EMBO J..

[2] Moren B (2012). EHD2 regulates caveolar dynamics via ATP-driven targeting and oligomerization. Mol. Biol. Cell.

[3] Torrino, S. *et al*. EHD2 is a mechanotransducer connecting caveolae dynamics with gene transcription. *J Cell Biol*, 10.1083/jcb.201801122 (2018). 10.1083/jcb.201801122 PMC6279385 30348749

[4] Li M (2013). Effects of EHD2 interference on migration of esophageal squamous cell carcinoma. Med. Oncol..

[5] Bignotti E (2006). Differential gene expression profiles between tumor biopsies and short-term primary cultures of ovarian serous carcinomas: identification of novel molecular biomarkers for early diagnosis and therapy. Gynecol. Oncol..

[6] Welinder C (2017). Correlation of histopathologic characteristics to protein expression and function in malignant melanoma. PLoS One.

[7] Liu J (2016). Decreased Expression of EHD2 Promotes Tumor Metastasis and Indicates Poor Prognosis in Hepatocellular Carcinoma. Dig. Dis. Sci..

[8] Yang X, Ren H, Yao L, Chen X, He A (2015). Role of EHD2 in migration and invasion of human breast cancer cells. Tumour Biol..

[9] Shi Y (2015). Decreased expression and prognostic role of EHD2 in human breast carcinoma: correlation with E-cadherin. J. Mol. Histol..

[10] Bray F (2018). Global cancer statistics 2018: GLOBOCAN estimates of incidence and mortality worldwide for 36 cancers in 185 countries. CA Cancer J. Clin..

[11] Perou CM (2000). Molecular portraits of human breast tumours. Nature.

[12] Foulkes WD, Smith IE, Reis-Filho JS (2010). Triple-negative breast cancer. N. Engl. J. Med..

[13] Harbeck N, Gnant M (2017). Breast cancer. Lancet.

[14] Morris SR, Carey LA (2007). Molecular profiling in breast cancer. Rev. Endocr. Metab. Disord..

[15] Aydiner A (2015). Metaplastic Breast Carcinoma Versus Triple-Negative Breast Cancer: Survival and Response to Treatment. Medicine.

[16] Bianchini G, Balko JM, Mayer IA, Sanders ME, Gianni L (2016). Triple-negative breast cancer: challenges and opportunities of a heterogeneous disease. Nat. Rev. Clin. Oncol..

[17] Campbell LM, Maxwell PJ, Waugh DJ (2013). Rationale and Means to Target Pro-Inflammatory Interleukin-8 (CXCL8) Signaling in Cancer. Pharmaceuticals.

[18] Singh JK, Simoes BM, Howell SJ, Farnie G, Clarke RB (2013). Recent advances reveal IL-8 signaling as a potential key to targeting breast cancer stem cells. Breast Cancer Res..

[19] Cai B (2013). Differential roles of C-terminal Eps15 homology domain proteins as vesiculators and tubulators of recycling endosomes. J. Biol. Chem..

[20] Lamaze C, Tardif N, Dewulf M, Vassilopoulos S, Blouin CM (2017). The caveolae dress code: structure and signaling. Curr. Opin. Cell Biol..

[21] Yeow I (2017). EHD Proteins Cooperate to Generate Caveolar Clusters and to Maintain Caveolae during Repeated Mechanical Stress. Curr. Biol..

[22] Inoue K (2000). Interleukin 8 expression regulates tumorigenicity and metastasis in human bladder cancer. Cancer Res..

[23] Kitadai Y (1999). Transfection of interleukin-8 increases angiogenesis and tumorigenesis of human gastric carcinoma cells in nude mice. Br. J. Cancer.

[24] Singh RK, Lokeshwar BL (2009). Depletion of intrinsic expression of Interleukin-8 in prostate cancer cells causes cell cycle arrest, spontaneous apoptosis and increases the efficacy of chemotherapeutic drugs. Mol. Cancer.

[25] Ha H, Debnath B, Neamati N (2017). Role of the CXCL8-CXCR1/2 Axis in Cancer and Inflammatory Diseases. Theranostics.

[26] Kim Y (2017). Prognostic implication of histological features associated with EHD2 expression in papillary thyroid carcinoma. PLoS One.

[27] Lamaze C, Torrino S (2015). Caveolae and cancer: A new mechanical perspective. Biomed. J..

[28] Lehmann BD (2011). Identification of human triple-negative breast cancer subtypes and preclinical models for selection of targeted therapies. J. Clin. Invest..

[29] Amadori D (1997). Cell proliferation as a predictor of response to chemotherapy in metastatic breast cancer: a prospective study. Breast Cancer Res. Treat..

[30] Prat A (2013). Prognostic significance of progesterone receptor-positive tumor cells within immunohistochemically defined luminal A breast cancer. J. Clin. Oncol..

[31] Wolff AC (2007). American Society of Clinical Oncology/College of American Pathologists guideline recommendations for human epidermal growth factor receptor 2 testing in breast cancer. J. Clin. Oncol..

[32] Vincent-Salomon A (2007). HER2 status of bone marrow micrometastasis and their corresponding primary tumours in a pilot study of 27 cases: a possible tool for anti-HER2 therapy management?. Br. J. Cancer.

[33] Bieche I (2001). Quantification of estrogen receptor alpha and beta expression in sporadic breast cancer. Oncogene.

[34] Revision of the standards for the assessment of hormone receptors in human breast cancer; report of the second E.O.R.T.C. Workshop, held on 16-17 March, 1979, in the Netherlands Cancer Institute. *Eur J. Cancer* **16**, 1513–1515, 10.1016/0014-2964(80)90064-x (1980). 10.1016/0014-2964(80)90064-x 6262087

[35] Langton BC (1991). An antigen immunologically related to the external domain of gp185 is shed from nude mouse tumors overexpressing the c-erbB-2 (HER-2/neu) oncogene. Cancer Res..

[36] Bieche I (1999). Real-time reverse transcription-PCR assay for future management of ERBB2-based clinical applications. Clin. Chem..

[37] Meseure D (2016). Expression of ANRIL-Polycomb Complexes-CDKN2A/B/ARF Genes in Breast Tumors: Identification of a Two-Gene (EZH2/CBX7) Signature with Independent Prognostic Value. Mol. Cancer Res..

[38] Hanley JA, McNeil BJ (1982). The meaning and use of the area under a receiver operating characteristic (ROC) curve. Radiology.

