# Supplementary material for: EHD2 is a Predictive Biomarker of Chemotherapy Efficacy in Triple Negative Breast Carcinoma
Source: Sci Rep. 2020 May 14;10:7998. doi: 10.1038/s41598-020-65054-5 (PMC7224205; doi:10.1038/s41598-020-65054-5)
Supplement: Supplementary file 1 — Supplementary Information. [file 41598_2020_65054_MOESM1_ESM.pdf]

Supplementary information for

EHD2 is a Predictive Biomarker of Chemotherapy Efficacy in Triple Negative Breast Carcinoma.

Wei-Wei Shen, Ivan Bièche, Laetitia Fuhrmann, Sophie Vacher, Anne Vincent-Salomon, Stéphanie Torrino* and Christophe Lamaze*

*** Correspondence to**: stephanie.torrino@unice.fr, christophe.lamaze@curie.fr

This PDF file includes:
Supplementary Table 1 to 4
Supplementary Figure 1

Supplementary figure 1: Uncropped blot of figure 2b

Supplementary Table S1: Characteristics of the 526 breast tumors used for RT-qPCR			
	**Number of patients (%)**	**Number with metastases (%)**	**p [a]**
Total	526 (100)	209 (39.7)	
**Age**			
≤50	124 (23.6)	51 (41.1)	0.59 (NS)
>50	402 (76.4)	158 (39.3)	
**SBR histological grade [b,c]**			
I	60 (11.7)	12 (20.0)	**0.0016**
II	240 (47.0)	99 (41.3)	
III	211 (41.3)	94 (44.5)	
**Macroscopic tumor size [e]**			
≤25mm	247 (47.9)	76 (30.8)	**<0.0001**
>25mm	269 (52.1)	132 (49.1)	
**Lymph node status [d]**			
0	159 (30.5)	48 (30.2)	**<0.0001**
1-3	249 (47.8)	87 (34.9)	
>3	113 (21.7)	72 (63.7)	
**ERa status**			
Negative	181 (34.4)	76 (42.0)	0.095 (NS)
Positive	345 (65.6)	133 (38.6)	
**PR status**			
Negative	255 (48.5)	110 (43.1)	**0.021**
Positive	271 (51.5)	99 (36.5)	
**ERBB2 status**			
Negative	396 (75.3)	152 (38.4)	0.15 (NS)
Positive	130 (24.7)	57 (43.8)	
**Molecular subtypes**			
RH- ERBB2-	102 (19.4)	38 (37.3)	0.050 (NS)
RH- ERBB2+	72 (13.7)	36 (50.0)	
RH+ ERBB2-	294 (55.9)	114 (38.8)	
RH+ ERBB2+	58 (11.0)	21 (36.2)	

[a] Log-rank test. NS : not significant

[b] Scarff Bloom Richardson classification.

[c] Information available for 511 patients.

[d] Information available for 521 patients.

[e] Information available for 516 patients.

Supplementary Table S2: Characteristics of the 423 breast tumors used to construct TMA

	Number of patients (%)	Number of patients with metastases (%)	*p* -value [a]
**TOTAL**	**423 (100)**	**65 (15.4)**	
**Age**			
≤50	158 (37.3)	24 (36.9)	0.94 (NS)
>50	265 (62.7)	41 (63.1)	
**Histological subtypes**			
Ductal carcinoma	416 (98.3)	62 (95.4)	0,077 (NS)
Other	7 (1.7)	3 (4.6)	
**SBR histological grade**			
I	66 (15.6)	5 (7.7)	**0.0077**
II	125 (29.6)	13 (20)	
III	232 (54.8)	47 (72.3)	
**Tumor size (mm)**			
pT1 (≤20)	277 (65.5)	25 (38.5)	**<0.0001**
pT2 (>20-≤50)	130 (30.7)	36 (55.4)	
pT3 (>50)	12 (2.8)	4 (6.1)	
pT4	4 (1)	0 (0)	
**Lymph node status**			
pNx (unknow	2 (0.5)	0 (0)	**<0.0001**
pN0	229 (54.1)	27 (41.6)	
pN1a	128 (30.3)	13 (20)	
pN2a	50 (11.8)	16 (24.6)	
pN3a	14 (3.3)	9 (13.8)	
**ER status**			
Negative	165 (39)	27 (41.5)	0.65 (NS)
Positive	258 (61)	38 (58.5)	
**PR status**			
Negative	194 (45.9)	39 (60)	**0.013**
Positive	229 (54.1)	26 (40)	
**ERBB2 status**			
Negative	340 (80.4)	59 (90.8)	**0.022**
Positive	83 (19.6)	6 (9.2)	
**Ki67 status**			
<14%	80 (18.9)	2 (3.1)	**0.0004**
≥14%	343 (81.1)	63 (96.9)	
**Molecular subtypes**			
Luminal A	125 (29.6)	8 (12.3)	**<0.0001**
Luminal B	110 (26)	28 (43.1)	
HER2+	83 (19.6)	6 (9.2)	
TNBC	105 (24.8)	23 (35.4)	

[a] Chi-squared test or Fisher test if applicable

Supplementary Table S3: Characteristics of the 101 triple negative breast tumors

	**Number of patients (%)**	**Number with metastases (%)**	***p*** [a]
Total	101 (100)	37 (36.6)	
Age			
≤50	38 (37.6)	15 (39.5)	0.66 (NS)
>50	63 (62.4)	22 (34.9)	
SBR histological grade [b,c]			
I	3 (3.2)	1 (33.3)	0.96 (NS)
II	18 (18.9)	8 (44.4)	
III	74 (77.9)	27 (36.5)	
Macroscopic tumor size			
≤25mm	39 (38.6)	11 (28.2)	0.14 (NS)
>25mm	62 (61.4)	26 (41.9)	
Lymph node status			
0	48 (47.5)	15 (31.3)	0.21 (NS)
1-3	39 (38.6)	14 (35.9)	
>3	14 (13.9)	8 (57.1)	

[a] Log-rank test. NS : not significant

[b] Scarff Bloom Richardson classification.

[c] Information available for 95 patients.

Supplementary Table S4: Characteristics of the 228 triple negative breast tumors			
	**Number of patients (%)**	**Number with metastases (%)**	***p*** [a]
Total	228 (100.0)	48 (21.1)	
Age			
≤50	84 (36.8)	16 (19.0)	0.52 (NS)
>50	144 (63.2)	32 (22.2)	
SBR histological grade [b,c]			
I	2 (0.9)	0 (0)	0.81 (NS)
II	23 (10.1)	5 (21.7)	
III	202 (89.0)	43 (21.3)	
Macroscopic tumor size			
≤25mm	148 (64.9)	25 (16.9)	**0.0088**
>25mm	80 (35.1)	23 (28.8)	
Lymph node status [d]			
0	153 (68.6)	19 (12.4)	**<0.0001**
1-3	40 (17.9)	11 (27.5)	
>3	30 (13.5)	17 (56.7)	

[a] Log-rank test. NS : not significant

[b] Scarff Bloom Richardson classification.

[c] Information available for 227 patients.

[d] Information available for 223 patients.

a

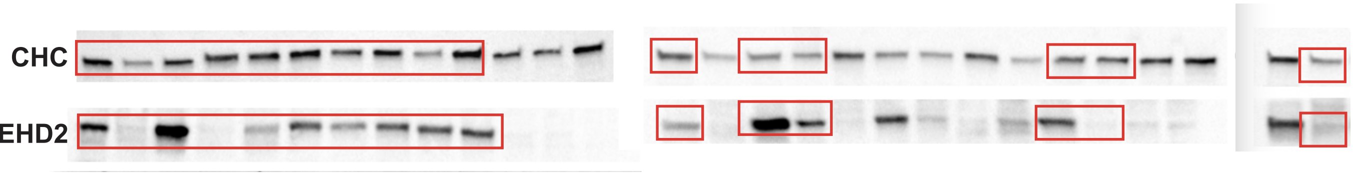

Supplementary Figure 1
